# Editorial

**Published:** 1895-12

**Authors:** 


### Editorial.

With this number, the SOUTHERN MEDICAL RECORD rounds the first quarter of a century of its existence. It has successfully combatted the perils that beset the way of medical journals, and begins its twenty-sixth year in sturdy and robust health and with the best prospects for continued prosperity.

It is deemed advisable to issue the journal on the first instead of the fifteenth of the month. This plan will be pursued in the future, beginning with the first day of the new year.

The subscription list has been largely increased during the year, which is a source of no little gratification to the management, as being a practical way of expressing appreciation of their efforts towards improvement of the journal. We confidently believe that advertisers will promote their business interests by considering this journal, as it reaches a large and increasing number of medical men in the South and Southwest and occupies a high position in the profession, as its twenty-five years of prosperity can attest.

We want brief, practical articles for publication, particularly from our own subscribers, and especially request that they will send them in. The report of an interesting case, or a therapeutic note, is always read, and the experience of the writer may help the reader in a difficult position.

Don't let your pen corrode. Put down your experiences on paper and thus embalm them forever. There is a doctor in an obscure little town in Louisiana, who has done this, and he is known all over the United States. Follow his example.

The RECORD wishes its readers a very merry Christmas.

---

SEE OUR GREAT PREMIUM OFFER

on Page 644. Any instrument or book on the list and the RECORD for one year for $2.00. Many of these instruments cannot be purchased in the stores at this price. They are from a well-known house, and are fully guaranteed. Make your selection, and send in your subscription at once.

